# Transcriptional oscillation of canonical clock genes in mouse peripheral tissues

**DOI:** 10.1186/1471-2199-5-18

**Published:** 2004-10-09

**Authors:** Takuro Yamamoto, Yasukazu Nakahata, Haruhiko Soma, Makoto Akashi, Takayoshi Mamine, Toru Takumi

**Affiliations:** 1 Osaka Bioscience Institute, Suita, Osaka 565-0874, Japan; 2 Life Science Laboratory, Materials Laboratories, Sony Corporation, Shinagawa, Tokyo 144-0001, Japan; 3 Graduate School of Medicine, Osaka City University, Osaka 545-8585, Japan

## Abstract

**Background:**

The circadian rhythm of about 24 hours is a fundamental physiological function observed in almost all organisms from prokaryotes to humans. Identification of clock genes has allowed us to study the molecular bases for circadian behaviors and temporal physiological processes such as hormonal secretion, and has prompted the idea that molecular clocks reside not only in a central pacemaker, the suprachiasmatic nuclei (SCN) of hypothalamus in mammals, but also in peripheral tissues, even in immortalized cells. Furthermore, previous molecular dissection revealed that the mechanism of circadian oscillation at a molecular level is based on transcriptional regulation of clock and clock-controlled genes.

**Results:**

We systematically analyzed the mRNA expression of clock and clock-controlled genes in mouse peripheral tissues. Eight genes (*mBmal1*, *mNpas2*, *mRev-erbα*, *mDbp*, *mRev-erbβ*, *mPer3*, *mPer1 *and *mPer2*; given in the temporal order of the rhythm peak) showed robust circadian expressions of mRNAs in all tissues except testis, suggesting that these genes are core molecules of the molecular biological clock. The bioinformatics analysis revealed that these genes have one or a combination of 3 transcriptional elements (RORE, DBPE, and E-box), which are conserved among human, mouse, and rat genome sequences, and indicated that these 3 elements may be responsible for the biological timing of expression of canonical clock genes.

**Conclusions:**

The observation of oscillatory profiles of canonical clock genes is not only useful for physiological and pathological examination of the circadian clock in various organs but also important for systematic understanding of transcriptional regulation on a genome-wide basis. Our finding of the oscillatory expression of canonical clock genes with a temporal order provides us an interesting hypothesis, that cyclic timing of all clock and clock-controlled genes may be dependent on several transcriptional elements including 3 known elements, E-box, RORE, and DBPE.

## Background

The circadian rhythm of about 24 hours is a fundamental physiological function observed in almost all organisms from prokaryotes to humans. Circadian rhythms have been known to be generated in pacemaker cells, the suprachiasmatic nuclei (SCN) of hypothalamus in mammals, and entrained by environmental cues, such as light, temperature, noise, feeding or social cues, whereas a recent analysis using mPer2^luciferase ^knockin mice has demonstrated that peripheral tissues express self-sustained circadian oscillations [1]. The output of circadian oscillation appears as locomotive activity, hormonal secretion, the sleep-wake cycle, and many other physiological functions. Disruption of the circadian rhythms has been associated with various kinds of diseases, such as cardiovascular diseases, psychiatric diseases and cancer in humans [2-6]. Identification of clock genes has allowed study of the molecular bases for circadian behaviors and temporal physiological processes and has prompted the idea that molecular clocks reside not only in a central pacemaker, but also in peripheral tissues, even in immortalized cells [2,3,6]. Furthermore, previous molecular dissection revealed that the mechanism of circadian oscillation at a molecular level is based on transcriptional regulation of clock and clock-controlled genes, which consists of interwoven positive and negative feedback loops [2,7-10].

There is a distinct connection between genes and behaviors in circadian rhythms, which is conserved from fly or other lower organisms to humans [6,8]. The *Drosophila *period mutants, originally identified as a circadian mutant brought us the first clock gene, *period *[8,11], while a point mutation of *hPer2 *was recently shown to cause a familial advanced sleep phase syndrome [12]. As described above, circadian rhythms rely on a negative feedback loop in gene expression that involves a limited number of clock genes. Recent molecular dissection has increased our understanding of the molecular nature of the transcriptional regulation of some clock genes. The circadian phenotypes at the cellular level may be represented as temporal mRNA expression. Global gene expression profiling using microarrays has led to the discovery of many circadian-regulated genes, but there is only a minor overlap of cycling transcripts between tissues [10,13,14]. Thus, circadian rhythms are an appropriate study target for systems biology.

In this study, we systematically examined the mRNA expression of common circadian-regulated genes in several mouse peripheral tissues and made oscillatory profiles of canonical clock genes. Moreover, by bioinformatics, we identified 3 clock elements for circadian transcription (E-box, RORE, DBPE). These 3 elements and their combination would suffice to explain the biological timing of expression of these clock and clock-controlled genes.

## Results and discussion

**Figure 1:**
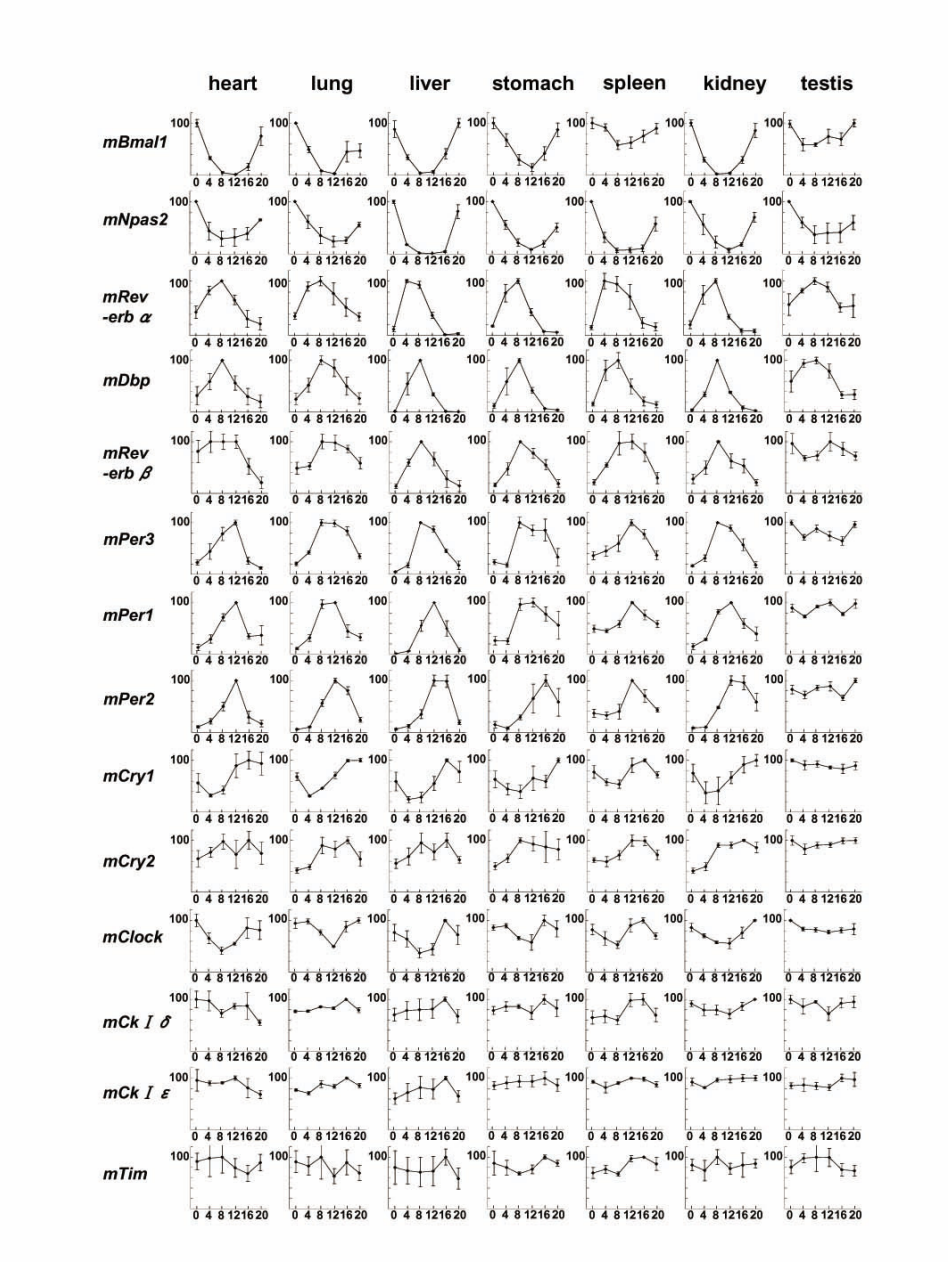
Temporal mRNA expression of clock and clock-related genes in mouse peripheral tissues Abscissa presents time at CT (circadian time); and ordinate, mRNA amounts. The relative levels of each RNA were normalized to the corresponding G3-PDH RNA levels. The maximum RNA amount was set to 100. Data are presented as the mean ± SE of triplicate samples.

**Figure 2:**
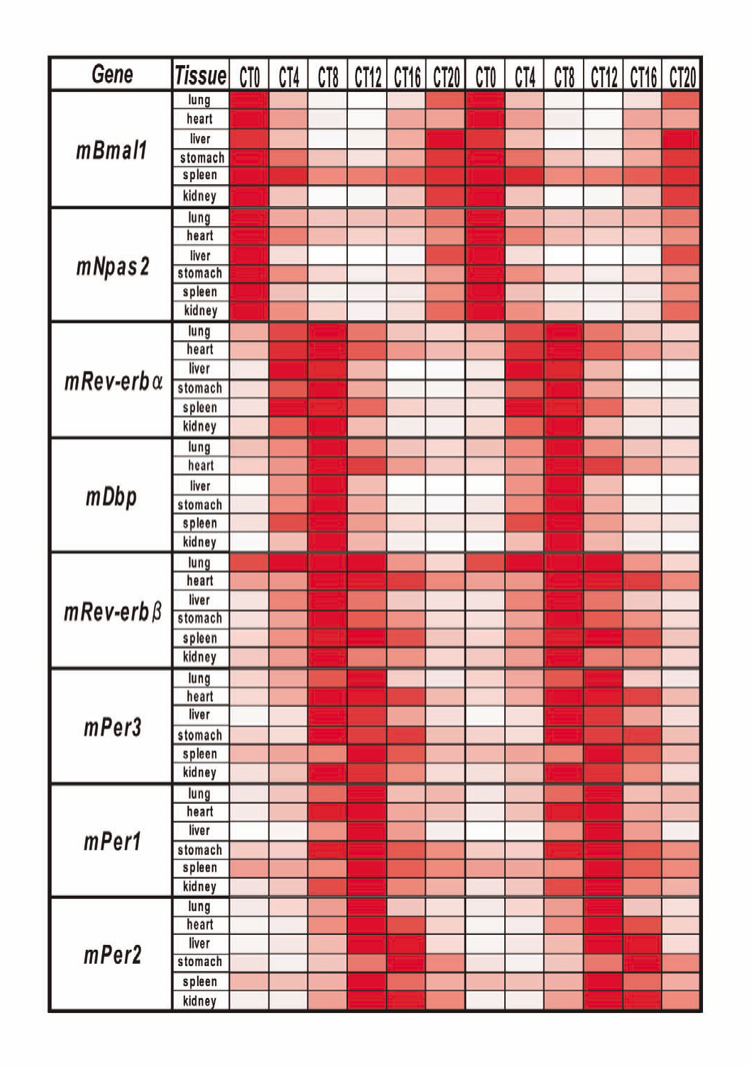
Circadian mRNA expression of canonical clock genes in mouse peripheral tissues The maximum RNA amount in a 24-h period is indicated by the darkest red (100), while no RNA (0) shown by white. The depth of the color corresponds to the RNA amount.

**Figure 4:**
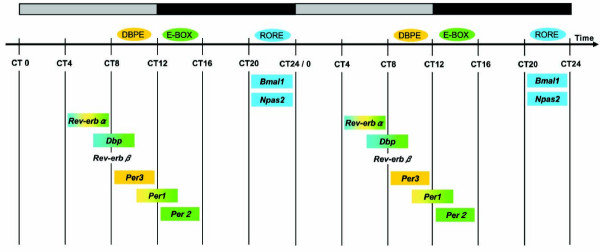
Diagram representing the relationship between 3 transcriptional elements and the peak mRNA expression of canonical clock genes The peak of *Per3 *mRNA expression is at CT8-12, and its regulatory region includes only conserved DBPEs (yellow) among the 3 elements. The peak of *Per2 *mRNA is at CT12-16, and its promoter region includes only an E-box (green). The peaks of *Bmal1 *and *Npas2 *mRNA are at CT20-0, and their regulatory regions contain only ROREs (blue). The peaks for the other clock genes, which contain 2 or 3 elements, are placed as indicated. The bar at the top represents light (gray) and dark (black) cycles.

To examine the circadian expression of mouse clock and clock-related genes in peripheral tissues, we performed the quantitative real-time reverse transcription-polymerase chain reaction (RT-PCR) method on mRNAs from 7 different mouse peripheral tissues (heart, lung, liver, stomach, spleen, kidney, and testis; Fig. 1). After entrainment of housed mice for 2 weeks under a light-dark (LD) cycle, samples were collected every 4 hr starting at circadian time (CT) 0 (n = 3 at each time point) in the third dark-dark (DD) cycle. Out of 14 mouse genes examined, 8 genes (*mBmal1*, *mNpas2*, *mRev-erbα*, *mDbp*, *mRev-erbβ*, *mPer3*, *mPer1 *and *mPer2*; given in the temporal order of the rhythm peak; see also Fig. 2) showed robust circadian of mRNA expression in all tissues except the testis. These genes should thus be considered to be core molecules of the circadian clock. Circadian mRNA expression patterns were similar in each tissue with the exception of testis, where weak or no rhythm was observed. This common pattern of the rhythm throughout many peripheral tissues implies that there may exist a universal mechanism for resetting the peripheral clock. The peak transcript level of each circadian rhythm was as follows: *mBmal1 *and *mNpas2*, in subjective night at CT20-CT0; *mRev-erbα*, in subjective day at CT4-8; *mDbp *and *mRev-erbβ*, at CT8; *mPer3*, at CT8-12; *mPer1*, at CT12; and *mPer2*, at CT12-16 (Figs. 1, 2, and 4). In the peripheral tissues mRNA peaks occurred approximately 4 hr later than those in the central pacemaker, SCN [15-20]. In the testis, 4 genes (*mBmal1*, *mNpas2*, *mRev-erbα*, and *mDbp) *showed weak rhythms of their mRNA expression, while no other genes, including the *mPer *family, showed any clear oscillation. Surprisingly, the expression of *mPer1 *transcripts in the testis, which did not show a circadian rhythm, was substantially higher than other tissues, and exceeded RNA expression of other genes studied in the testis. This is consistent with data recently reported [21-23]; although a previous report indicated circadian rhythm of *mPer1 *in the testis [20]. These findings suggest that *mPer1 *may play an alternative role in the testis, including developmental regulation during spermatogenesis. The rhythm of *mCry1 *mRNA expression was obviously circadian (peaking at CT16-20, with a trough at CT4-8) except in the testis, but the peak-trough amplitude was relatively smaller than that of the above genes. *mCry2 *and *mClock *RNA levels seemed to be rhythmic except in the testis, but the rhythm was rather weak and not clearly circadian. No circadian rhythms were observed in the remaining 3 genes examined, i.e. *mCKI-δ*, *mCKI-ε*, and *mTim*. Despite a central role in *Drosophila *for *timeless *(*dTim*), the mammalian homologue (*mTim*) was originally found to show weak or no rhythm in the SCN [24-26]; and its functional role in mammalian clocks remains controversial [27,28].

**Figure 3:**
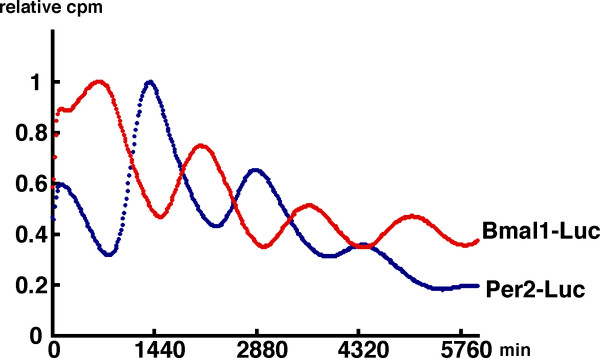
Transcriptional oscillation of *Bmal1 *and *Per2 *Transcriptional oscillation of *Bmal1 *(red) and *Per2 *(blue) was monitored by using a cell culture-based luminescence reporter assay. NIH3T3 cells were transfected with the *hBmal1 *-Luc or *mPer2 *-Luc constructs and then stimulated with a high concentration of serum. After the serum shock, in the presence of luciferin, light emission was measured and integrated for 1 min at intervals of 15 min. Ordinate and abscissa represent relative counts and time after serum shock, respectively. The peak of the count was set to 1. 1440 minutes = 1 day.

As described above, the expression of 8 genes fluctuated in an overt circadian fashion; and so we aligned them in the order of the peak of their oscillatory phase (Fig. 2). Among them, the transcriptional oscillation of 2 representative clock genes, *Bmal1 *and *Per2*, was examined by using the real-time luciferase reporter assay. NIH3T3 cells were transfected with the *hBmal1*-Luc or *mPer2*-Luc construct and then stimulated with a high concentration of serum. After the serum shock, in the presence of luciferin, light emission was measured and integrated for 1 min at intervals of 15 min. Both promoters fused to luciferase showed circadian rhythms (Fig. 3). The phase of *Bmal1 *oscillation in cultured cells was almost the opposite of that of *Per2*, which is consistent with data of mRNA expression in mouse peripheral tissues obtained by real-time RT-PCR (Fig. 2). This result indicates that the promoter regions used in the real-time luciferase reporter assay are sufficient for producing circadian transcriptional oscillation. The promoter analyses of several clock genes have been reported, as described below.

**Figure 5:**
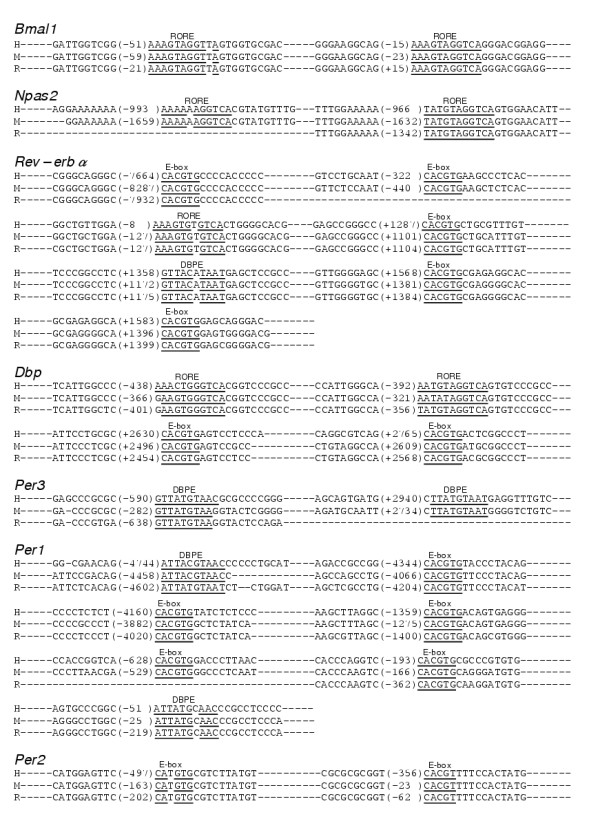
Table 1. Three transcriptional elements (RORE, E-box, and DBPE) conserved among the sequences of 3 species (human, mouse, and rat) Numbers in parenthesis indicate the number from the transcriptional start site. The sequences of the binding elements are in bold type, and the sequences matching the consensus sequence are underlined. H: human, M: mouse, R: rat.

A molecular mechanism of canonical clock genes is based on transcriptional regulation via interlocked feedback and/or feed-forward loops [2,4,7,8]. One example of the regulation of a known characterized gene in mammals is that of *Per1*. The transcription of *Per1 *is activated by binding of the CLOCK/BMAL1 hetero-complex, both members of which are bHLH-PAS (basic helix-loop-helix-Per-Arnt-Sim) proteins, to the E-boxes in the promoter region of *Per1 *[29]. The translated PER1 is posttranslationally modified by CKI-ε [30] and, together with other clock proteins such as CRYs [31], is returned to the nucleus to suppress its own transactivation, resulting in closure of the PER1 loop. E-box elements are also known to be essential for transcriptional regulation of many clock-controlled output genes including the vasopression genes [32]. On the other hand, one of the positive elements, BMAL1, whose mRNA expression is cycled antiphase to *Per*s, as described above, forms another loop [33,34]. The orphan nuclear receptors RORα and REV-ERBα regulate circadian transcription positively and negatively, respectively, through ROR/REV-ERB elements (ROREs) in the promoter region of *Bmal1 *[16,35]. Moreover, an *in silico *search identified these 2 ROREs in the promoter region of *Npas2 *(Table 1, see figure 5), the expression pattern of which was very similar to that of *Bmal1*. These findings gave us the idea that the circadian pattern of RNA expression might be dependent on transcriptional regulation by specific transcription factors.

Using the NCBI database and Celera Database System, we systematically searched for the above 2 elements and another clock element, a DBP-binding element (DBPE), described below. These elements are conserved among human, mouse, and rat genome sequences in the regions 9-kb upstream and 5-kb downstream of the transcription start site (Table 1, see figure 5). Intriguingly, the transcriptional elements corresponded to the cyclic pattern of the clock genes shown in Figure 4. *Bmal1 *and *Npas2*, the circadian peaks of which were both in subjective night at CT20-24, included ROREs in their promoters, as described above. In the promoter of *Per1 *and *Per2*, the peaks of which were in subjective day at CT12-16, E-boxes were found. In fact, the conserved E-box in *Per2 *is not a typical E-box of the molecular clock, CACGTG, but an atypical element, CACGTT. Compared with *Per1*, which contains 5 conserved E-boxes, *Per2 *may have other unknown factors and elements responsible for its robust transcriptional oscillation. *Per1*, whose peak was a bit earlier than that of *Per2*, has another element, a DBPE, in its promoter region, in addition to the 5 E-boxes well studied *in vitro *[36]. *Per3*, the peak of which was even earlier at CT8-12, did not have conserved E-boxes but instead contained DBPEs in its gene. Three genes, *Rev-erbα*, *Dbp*, and *Rev-erbβ*, the peaks of which were between those of *Bmal1 *and *Per*, had a mixed combination of the elements. The *Rev-erbα *genome sequence included 1 RORE, 1 DBPE, and 5 E-boxes, whereas *Rev-erbβ *included all 3 elements only in the mouse genome sequence. *Dbp *contained 2 ROREs and 2 E-boxes in each genome. Among the elements described above, some of them in (*Bmal1*, *Dbp*, and *Per1) *were experimentally studied and confirmed [16,29,35-38].

Transcripts of 8 genes (*mBmal1*, *mNpas2*, *mRev-erbα*, *mDbp*, *mRev-erbβ*, *mPer3*, *mPer1*, and *mPer2*) showed a robust circadian rhythm in different peripheral tissues (see Fig. 1). The amount of mRNA in the trough was nearly zero and the peak-trough amplitude of these genes was clearly higher than that of the others examined. Thus, in terms of mRNA expression among the canonical clock genes examined, these 8 genes likely constitute the core molecules of a molecular circadian clock. The expression timing in a 24-h period appears to be conveyed through 3 kinds of sequence elements bound by specific transcription factors (see Fig. 4). Recent genome-wide analyses using microarrays revealed that many genes (about 10 % of the total number of genes studied) oscillated but only several tens of common genes overlapped between two tissues examined [13,14]. Among the core candidate genes with similar circadian regulation in those 2 tissues, we examined the circadian transcription of 15 candidate genes besides the known clock genes, but could not find genes with oscillatory behavior in different peripheral tissues comparable to that in the 8 genes described above. The 8 genes studied here may approximate the entirety of the core oscillatory genes in the genome. If so, the 3 elements described here may be sufficient for explaining the biological timing of mRNA expression of clock genes. However, our preliminary results showed that an atypical E-box in *Per2 *promoter may be insufficient for full transcriptional oscillation (Akashi and Takumi, unpublished data). Further detailed studies of each promoter, combined with systematic analyses using microarrays and real-time RT-PCR, will give us a more detailed comprehension of the intertwined positive and negative regulatory loops of molecular biological clocks.

## Conclusions

The current study has clarified the detailed circadian expression of mRNAs for clock and clock-related genes in different peripheral tissues of the mouse. The observation of oscillatory profiles of canonical clock genes is not only useful for physiological and pathological examination of the circadian clock in various organs but also important for systematic understanding of transcriptional regulation on a genome-wide basis. Our finding of the oscillatory expression of canonical clock genes in a temporal order provides us an interesting hypothesis, that cyclic timing of all clock and clock-controlled genes may be dependent on several transcriptional elements including 3 known elements, E-box, RORE, and DBPE.

## Methods

### Animals

Male Balb/c mice purchased 5 weeks postpartum from Japan SLC (Hamamatsu, Japan), were exposed to 2 weeks of light-dark (LD) cycles and then kept in complete darkness as a continuation of the dark phase of the last LD cycle. mRNA expression was examined in the third dark-dark (DD) cycle. All protocols of experiments using animals in this study were approved by the OBI (Osaka Bioscience Institute) Animal Research Committee.

### Quantitative RT-PCR

Real-time quantitative RT-PCR was performed by using an ABI PRISM 7000 (Applied Biosystems). The PCR primers were designed with Primer Express software (Applied Biosystems), and the sequences of the forward and reverse primers were as follow: *mPer1 *FW: CAG GCT AAC CAG GAA TAT TAC CAG C, *mPer1 *RV: CAC AGC CAC AGA GAA GGT GTC CTG G; *mPer2 *FW: GGC TTC ACC ATG CCT GTT GT, *mPer2 *RV: GGA GTT ATT TCG GAG GCA AGT GT; *mPer3 *FW: CTG CTC CAA CTC AGC TTC CTT T, *mPer3 *RV: TTA GAC AGC AAG GCT CTG GTT CT; *mNpas2 *FW: GTA TGC ACA GAG CCA AGT GAT GTT, *mNpas2 *RV: TGC TCA CTG TGC AGA GAT GTT G; *mDbp *FW: AAT GAC CTT TGA ACC TGA TCC CGC T, *mDbp *RV: GCT CCA GTA CTT CTC ATC CTT CTG T; *mBmal1 *FW: GCA GTG CCA CTG ACT ACC AAG A, *mBmal1 *RV: TCC TGG ACA TTG CAT TGC AT; *mRev-erbα *FW: CGT TCG CAT CAA TCG CAA CC, *mRev-erbα *RV: GAT GTG GAG TAG GTG AGG TC; *mRev-erbβ *FW: ACG GAT TCC CAG GAA CAT GG, *mRev-erbβ *RV: CCT CCA GTG TTG CAC AGG TA; G3-PDH FW: ACG GGA AGC TCA CTG GCA TGG CCT T, G3-PDH RV: CAT GAG GTC CAC CAC CCT GTT GCT G; *mCry1 *FW: CCC AGG CTT TTC AAG GAA TGG AAC A, *mCry1 *RV: TCT CAT CAT GGT CAT CAG ACA GAG G; *mCry2 *FW: GGG ACT CTG TCT ATT GGC ATC TG, *mCry2 *RV: GTC ACT CTA GCC CGC TTG GT; *mCKIε *FW: GGA TGT GAA GCC CGA CAA CTT, *mCKIε *RV: TCT CGA CGG CTT TGC TCA AT; *mCKIδ *FW: CCA GCC TGG AAG ACC TGT TC, *mCKIδ *RV: TGG CCA GCC CAA AGT CAA; *mClock *FW: CCT ATC CTA CCT TGG CCA CAC A, *mClock *RV: TCC CGT GGA GCA ACC TAG AT; *mTim *FW: ACA TGT GGG CAA TGG CTT, *mTim *RV: CTG CTC CAC AAA GTG AAA GGT. Specificity of gene amplification was confirmed by measuring the size and purity of the PCR product by gel electrophoresis, and by analyzing the dissociation curve with ABI PRISM 7000 SDS software (Applied Biosystems). For a 25-μl PCR reaction, 50 ng cDNA template was mixed with the forward and reverse primers to a final concentration of 300 nM each and 12.5 μl of 2x SYBR Green PCR Master Mix (Applied Biosystems). The reaction was first incubated at 50°C for 2 min, then at 95°C for 10 min, followed by 40 cycles of 95°C for 15 sec and 60°C for 1 min. Each gene-specific PCR was performed in triplicate. G3-PDH primers were used as the control.

### Real-time luciferase reporter assay

NIH3T3 cells were cultured, transfected with *hBmal1 *-Luc or *mPer2 *-Luc, and incubated for 24 hours. The medium was then exchanged for serum-rich medium (DMEM, supplemented with 50 % serum). Two hours later this medium was replaced with normal culture medium. In the presence of 0.1 mM luciferin, light emission was measured and integrated for 1 min at intervals of 15 min, with a photomultiplier tube (Hamamatsu Photonics).

### *In silico *search

The sequences were downloaded from the Celera Database System and the NCBI Gene database. Each gene sequence spanning from 9 kb upstream to 4 kb downstream of the transcription start site was examined in each database. Multiple sequence alignments of these sequences for each gene were obtained by Clustalw version 1.83 with default parameters. The binding elements were then searched from these alignments using a pattern finding tool, fuzznuc, with the following consensus sequences allowing for a 1-base mismatch:

DBPE: [GA]T[GT]A[TC]GTAA[TC]

E-Box: CACGTG

RORE: [AT]A[AT]NT[AG]GGTCA

The accession numbers used and the sequence numbers analyzed are as follow: *Bmal1*; Human, NT_009237.16, 12054318–12069318, Mouse, NT_081129.1, 107781–122781, Rat, NW_047562.1, 13774073–13789073, *Npas2*; Human, hCG27614, 95632226–65646226, Mouse, mCG8437, 35980102–35994102, Rat, rCT22431, 39204499–39218499, *Rev-erbα*; Human, hCG93862, 34926094–34912094, Mouse, mCG15360, 105438925–105424925, Rat, rCG33292, 82492796–82478796, *Dbp*; Human, NT_011109.15, c21417778–21402778, Mouse, NT_078442.1, 59711–74711, Rat, NW_047558.1, 5120734–5135734, *Per3*; Human, NT_021937.16, 1962822–1977822, Mouse, NT_039268.2, c4331528–4316528, Rat, NW_047727.1, c8016956–8001956, *Per1*; Human, NT_010718.14, c6905708–6890708, Moues, NT_039515.2, 65661216–65676216, Rat, rCG34390, 52960430–52974430, *Per2*; Human, NT_005120.14, c5136562–5121562, Mouse, NT_039173.2, c5833757–5818757, Rat, NW_047817.1, c6827703–6812703.

## List of Abbreviations used

SCN, suprachiasmatic nuclei; RT-PCR, reverse transcription-polymerase chain reaction; CT, circadian time; LD, light-dark; DD, dark-dark; bHLH-PAS, basic helix-loop-helix-Per-Arnt-Sim; RORE, ROR/REV-ERB element; DBPE, DBP-binding element; NCBI, the National Center for Biotechnology Information.

## Authors' contributions

TY and YN carried out the molecular biology studies. HS carried out the in silico study. MA carried out the real-time luciferase reporter assay. TM participated in the coordination, and provided financial support. TT conceived of the study, participated in its design and coordination, and drafted the manuscript. All authors read and approved the final manuscript.
